# What Dominates the Female Class Identification? Evidence From China

**DOI:** 10.3389/fpsyg.2021.627610

**Published:** 2021-02-22

**Authors:** Peng Cheng, Jing Zhou, Ping Jiang, Zhijun Zhang

**Affiliations:** ^1^School of Resource and Environmental Sciences, Wuhan University, Wuhan, China; ^2^School of Public Administration of Guangxi University, Nanning, China; ^3^Key Laboratory of Urban Land Resources Monitoring and Simulation, MNR, Shenzhen, China

**Keywords:** female class, gender equality, psychological identification, ordered logit model, China

## Abstract

In advocating gender equality today, we should not only pay attention to women's social status but also call for the women's psychological identification of class equality. What dominates female class identification? To answer this question, based on the data of the Chinese General Social Survey (CGSS) in 2015, this study constructs a female class identity framework from five aspects: the mother's intergenerational influence, female personal characteristics, lifestyle, gender consciousness, and spouse status. In this study, the ordered logit model is used to empirically analyze the impact of various factors on female class identification, and the results show the following: (1) gender consciousness has a significant impact on female class identification. (2) Lifestyle has a significant impact on the situation of having a spouse. (3) Spouse status has a significant positive effect on female class identification. But (4) the mother's intergenerational influence has no effect on female class identification. Therefore, this paper suggests that we should improve laws and regulations to protect women's normal rights, encourage women to establish an independent and self-improvement character, and enhance the class consciousness of women, especially rural women, in order to achieve the overall improvement of female class and psychological identification.

## Introduction

Gender differences in social status have existed for a long time (Ciabattari, 2001; Mensah and Adjei, 2020; Takahashi et al., 2020). In primitive society, due to the difference in body shape and strength between men and women, men were better able to undertake the power-type labor. The distribution of housework between the husband and wife was uneven, and the status of women became increasingly lower over time (Nyman et al., 2018). Class status refers to the different social class levels, which are represented by a series of social status indicators, such as the educational level and income of the subject (Zajacova, 2006; Curtis, 2016; Zhao and Zhou, 2017). In China, since the 1990s, people have been arguing endlessly on the issues of “having a good marriage is more important than having a good job,” “the ultimate significance of women is to find a good spouse to get married and nourish children,” and “having a good marriage, and you can reduce 20 years of struggling.” Besides, under the concept of gender inequality, women's rights and interests are difficult to be protected, which affects women's values and class identity (Weeks, 1989). Influenced by the development of market economy background at that time, some women think that they can change their destiny by choosing to “marry successfully” instead of individual efforts, and attach their values to their spouses rather than themselves (Ye and Zhao, 2018).

In the past few decades, the proportion of women in the political institutions of almost every country in the world has increased significantly, and the trend of women's labor force participation has also gradually increased; the social status of women has been greatly improved compared with that in the past (Fitzsimmons et al., 2014; Uunk, 2015; Wu and Zhou, 2015; Hessami and da Fonseca, 2020; Koburtay et al., 2020). Under the background of economic transformation in China, women's social class is gradually stable in the constant changes (Chen and Ge, 2018). Recently, the education level of Chinese women has been increasing. According to the data of China Women's Development Program (2011–2020) in 2019 released by China Statistics Bureau, the number of female postgraduate students in China reaches 1.448 million, accounting for 50.6% of all postgraduate students. This data confirms the progress of women in receiving equal education, and the importance of women's voice in scientific research (Burt, 2019). However, has women's identification with their own class changed? Regarding the meaning of the phrase “class identification,” through literature review, we can find that scholars agree with the views of Mr. and Mrs. Jackman (Jackman and Jackman, 1973): class identification is the individual's perception of their own social class structure. Therefore, the existing research mainly defines “class identification” as comprising the following actions: each social member will measure or judge his social status according to his or her own conditions (such as economic strength and his own power) and will belong to a certain level of society (Anthias, 2001). The objective social status will affect the subjective class identity (Curtis, 2016). However, the improvement of the objective social status does not always involve the improvement of self-identity in the subjective sense, as there are still significant differences between them (Sakurai et al., 2010; Leicht et al., 2017; Koburtay et al., 2020). Gender inequality between men and women comprises not only the actual inequality but also the psychological inequality. In today's advocacy of gender equality, we should not only pay attention to women's social status but also to the appeal of women's psychological identification with class equality, which is more conducive to social stability, health, and sustainable development (Bolzendahl and Myers, 2004; Stevens and Martell, 2019; Patterson et al., 2020).

The research on class identification has been relatively extensive and has involved psychology, sociology, economics, gender research, and other research fields. Moreover, experts and scholars have different research perspectives on class identification, and the measurement standards have not been completely consistent. These scholars have mainly studied the influence of the social environment and personal characteristics on class identification (Zajacova, 2006; Sakurai et al., 2010). However, the class identification of female groups has gradually attracted attention. Female class identification not only emphasizes women's recognition of themselves but also reflects whether women attach importance to gender equality (Bolzendahl and Myers, 2004). Some scholars pay attention to the relationship between socioeconomic status and female class identification and believe that the higher the social status of women is, the higher their class identification is (Mendelson et al., 2008; Chen et al., 2020). There are also studies that focus on married women as the main object of study in order to analyze whether their class identification mainly comes from themselves or their spouses (Baxter, 1994; Bolzendahl and Myers, 2004). In addition, additional studies have focused on the differences between the class identification of urban and rural women. It has been found that compared with that of urban women, the sense of class identification of rural women is generally lower and that rural women are more dependent on their spouses (Beetham, 1998; Michelson, 2007; Bryant and Pini, 2009; Baylina and Rodó-Zárate, 2020). Regardless of whether the study focuses on the determination of whether married women's class identification comes from themselves or their spouses or focuses on the differences in class identification between urban and rural women, all of these studies provide important insights for further research on the index factors influencing female class identification.

**Figure 1:**
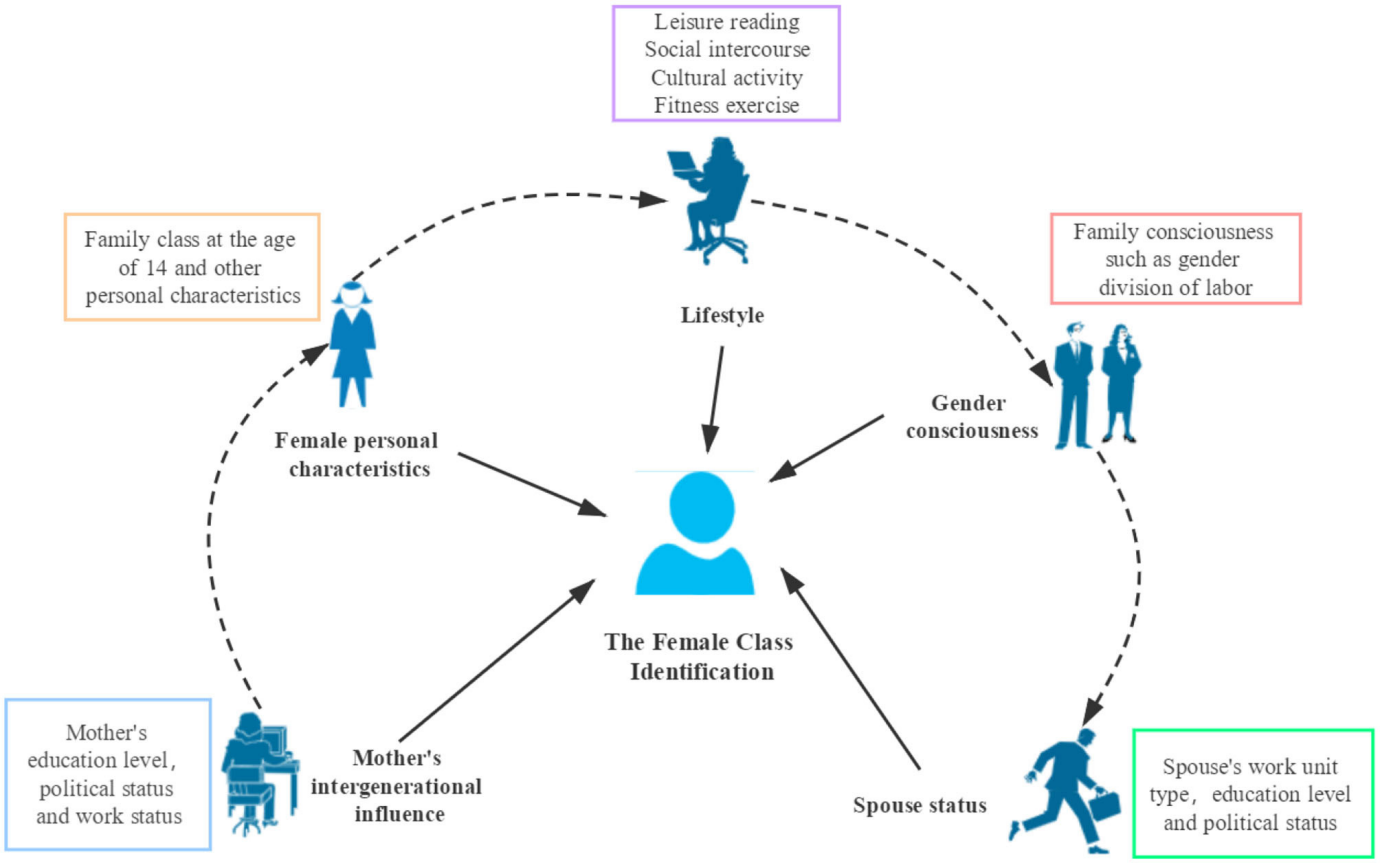
The framework of female class identification. *Source*: Author's own construction from the research framework.

However, these studies mainly focus on one aspect of the influencing factors of female class identification, and there are few comprehensive female class identification studies that examine the topic from the perspective of the young to middle-aged personal growth experience. In particular, there are few studies on the impact of mothers on the class identification of the next generation of females and on the impact of lifestyle on female class identification. This paper creatively puts forward the female class identification framework (Figure 1), which mainly includes the mother's intergenerational influence, female personal characteristics, lifestyle, gender consciousness, and spouse status. This paper summarizes some experiences that may have an important impact on women's own class identification in the process of women's growth and analyzes whether these factors truly affect female class identification and the degree of influence. Therefore, to provide a theoretical and practical reference for further promoting gender equality and female class identification, this study constructs an econometric ordered logit model and uses the data of the China General Social Survey (CGSS) obtained in 2015 in order to study the influencing factors of women's class identification.

## Literature Review

Class identification is an important research field of social development stratification (Zipp and Plutzer, 1996; Curtis, 2016; Varela et al., 2020). Many scholars have focused on the status of the objective social class in the past. Now, scholars have begun to pay attention to the differences in subjective class identification. In the past, the subjects of the study have mainly been urban residents and migrant workers, and these studies have mainly explored the impact of a series of factors, such as life experience, administrative level, the father's professional reputation, and housing consumption, on class identification (Zhao and Ge, 2014; Wang, 2017; Rubin and Stuart, 2018; Reeves, 2019; Lee et al., 2020). Regarding the focus of the research, which is advocacy related to pursuing equality between men and women, the current research object is mainly female groups, the analysis of which may improve to a certain degree of research significance the understanding of the class identity of female groups (Bolzendahl and Myers, 2004). Women's class identification is a kind of perception or feeling of their own position in the social class structure (Zipp and Plutzer, 1996; Rubin and Stuart, 2018). Baxter (1994) points out that the women's class perception is influenced by the state's gender openness and gender equality. This phenomenon is reflected in a series of female groups comprising rural women. The investigation and research reveals that rural women's class identification comes more from their own working conditions, the education level of their mothers and themselves, and the participation of the community, as well as the interpersonal communication with the people inside and outside the village (Michelson, 2007; Wang, 2017). Therefore, we can define the following as factors that affect women's class identification: gender consciousness, intergenerational influence of mothers, lifestyle, and spouse status.

In the family, the core unit of society, there is strict gender division in the division of labor. Generally, in terms of the family division of labor, women are mainly responsible for housework, while men are mainly responsible for going out to work (Besamusca et al., 2015; Bloom et al., 2015). In addition, most men still hope that women can play the traditional role of a good wife and good mother, and this expectation has an important impact on women's gender consciousness (Corrigall and Konrad, 2007; Pedersen, 2016), which will also affect women's own class identification. Some studies have found that women's self class identification is greatly affected by family because women usually associate their self-worth with their husbands (Zipp and Plutzer, 1996; Peake and Harris, 2002; Zajacova, 2006; Ye and Zhao, 2018). In particular, after distinguishing between urban and rural areas, age and education level, it is found that the traditional cognition of married women on their own class status will not change significantly because they live in the city, are young and have higher education. Even when their income and education level are higher than that of their husbands, their psychological attachment to their husbands will not be fundamentally changed (Baxter, 1994). In addition, women's subordination psychology may lead them to think that their spouse's class is more important than their own. In addition, because of the failure of these women to form their own independent class identification, these women think that their spouse's class determines to a great extent their own class identification (Zipp and Plutzer, 1996; Zajacova, 2006).

Class identification is also based on the concept of mobility and network. The class identity of contemporary people will be affected by the previous generation. When the upper generation's class identification is low, then the contemporary generation's class identification will also be low. In the process of women's growth, their mothers play an irreplaceable role in establishing the women's image and promoting class identification (Heath and Tan, 2018; Baylina and Rodó-Zárate, 2020; Kong et al., 2020). Some studies have shown that the mother's education experience, family economic level and objective class level have a significant impact on the women's perception of class mobility (Liu et al., 2013; Ziv and Arbel, 2020). With all kinds of information pouring in, individuals are no longer limited to uninteresting work and pay more attention to forms of entertainment. The members of each class show their relationship with and distance from other classes through the choice of interest (Chen et al., 2020). Taking as an example the investigation and research on the sports consumption psychology of different social classes of people, the results show that with the improvement of the social strata, the sports consumption attitude tends to be stronger (Schinke et al., 2019). When scholars take the middle class as the research object, they find that the unique taste shown in leisure tourism consumption has become the label with which individuals identify themselves, and the expression of this taste is a way for them to distinguish themselves from other people (Aramayona and García-Sánchez, 2019).

The literature review reveals that the existing literature on class identification, especially female class identification, mainly focuses on one aspect of the influencing factors. There is no comprehensive study that focuses on the four factors, namely, gender consciousness, the intergenerational influence of mothers, lifestyle, and spouse status, in order to conduct a long-term study of women's class identification. This type of comprehensive study is the innovation of this paper. Class identification is not just affected by one aspect but is constantly changing due to different experiences. It is necessary to comprehensively consider and analyze the real influencing factors of female class identification.

Therefore, to provide a certain reference for future research on female identification, this paper will use the CGSS data obtained in 2015 to study the influence of gender consciousness, mother's intergenerational influence, lifestyle, and spouse status on female class identification. Real gender equality not only exists because women can enjoy privileges but also because they can define their own class self-identification. Such recognition is not defined by anyone else or any external factors. This study is of great significance to the rise in women's status in all walks of life, and more importantly, to the pursuit of a more equal and harmonious social environment.

## Proposed Research Hypothesis

### The Hypothesis Between Gender Consciousness and Female Class Identification

Gender consciousness is an important aspect of the research on gender relations and mainly includes a series of indicators (Bolzendahl and Myers, 2004; Fagertun, 2017; Nyman et al., 2018), such as the gender division of labor, the difference in ability between men and women, marriage consciousness, and gender discrimination in employment. The statistics on these indicators are included in the CGSS data obtained in 2015. These views in the research on gender consciousness show that women agree with the low status of women in the relationship between men and women. Therefore, we propose hypothesis H1: the more women accept the view that women's status is low, the lower their sense of class identification is.

### The Hypothesis Between Mother's Intergenerational Influence and Female Class Identification

In the research on family relationships, studies have found that the mother has an important influence on the daughter's family outlook and values (Liu et al., 2013; Heath and Tan, 2018; Baylina and Rodó-Zárate, 2020; Kong et al., 2020). In daily life, if the family relationship is long-term unequal, i.e., if the father is stronger and the mother is always the weaker side, this situation is likely to lead to the formation of a child's view of discrimination against women. In rural areas, for example, girls may be easily overlooked and thus have less access to education (Kong et al., 2020). As time goes on, women who have no opportunity to receive education may still neglect their daughter's education in the next generation, which leads to the lower class identification of women in the next generation. Therefore, we propose hypothesis H2: the higher the social status of the mothers is, the stronger the social identification of women.

### The Hypothesis Between Lifestyle and Female Class Identification

Maslow's theory, from the lower level to higher levels, divides needs into five categories: physiological needs, security needs, social needs, respect needs, and self-realization needs (Noltemeyer et al., 2012). When individuals can basically meet their own survival needs, they will pursue higher-level needs. The higher the level of the residents' need is, the more likely it is that the residents' life is not limited to work, and the more they enjoy life (Kulich et al., 2017; van Breen et al., 2017; Rubin and Stuart, 2018). In contrast, after satisfying our basic postnatal tastes, individuals will have a better sense of happiness. As the residents satisfy their needs at one level, does the residents' class identification also improve at the same time? Therefore, we propose hypothesis H3: Among women, the higher their taste of life is, the higher their class identification is.

### The Hypothesis Between Spouse Status and Female Class Identification

In the division of the class identification in the husband and wife relationship, one kind of relationship is a “dependent type,” that is, one in which the female completely relies on the spouse to determine her own class (Pedersen, 2016). It is undeniable that whether a woman is married or not has a great influence on her life state. After marriage, women are more likely to devote their energy to their families, and the quality of the spouse's class will also affect women's class identification. More importantly, in reality, the way in which the work unit of a female's spouse is viewed may determine the spouse's degree of recognition in the society, thus affecting women's social identification. Therefore, we propose hypothesis H4: Among married women, the better the spouse's work unit is, the higher the female's class identification is.

## Data Source and Variable Setting

### Data

The data used in this study were obtained from the Chinese General Social Survey (CGSS, data sources: http://cgss.ruc.edu.cn) carried out in 2015 and sponsored by the China Social Science Foundation. The data were originally collected by Renmin University of China and Academic Institutions in China (Wang et al., 2020). By screening the control variables and eliminating the invalid questionnaires, 4,702 valid female samples were obtained.

### Variable Setting

In this study, all variables are divided into explained variables, explanatory variables, and control variables. To facilitate reading and checking, the code of each variable is consistent with that in the CGSS2015 questionnaire. Through sorting, the basic information of each variable is shown in Table 1.

**Table 1:** Assignment and descriptive statistics of the variables. *Source*: Author's own elaboration from the CGSS2015 dataset.

**Variable type**	**Indexes**	**Assignment**	**Average value**	**Standard deviation**
Explained variables	Female class identification (a431)	Lower class = 1; middle class = 2; upper class = 3	1.749	0.485
Explanatory variables	*Gender consciousness*			
	Gender division of labor consciousness (a421)	In total, disagree = 1; fully agree = 5 (increase in order)	3.294	1.484
	Gender ability consciousness (a422)	In total, disagree = 1; fully agree = 5 (increase in order)	2.934	1.521
	Marriage consciousness (a423)	In total, disagree = 1; fully agree = 5 (increase in order)	3.070	1.641
	Gender discrimination in employment (a424)	In total, disagree = 1; fully agree = 5 (increase in order)	1.818	2.147
	Housework distribution consciousness (a425)	In total, disagree = 1; fully agree = 5(increase in order)	3.787	1.388
	*Mother's intergenerational influence*			
	Mother's education level (a90b)	None = 0; primary school = 1; junior high school = 2; senior high school = 3; undergraduate = 4; postgraduate = 5	0.595	0.915
	Mother's political status (a90c)	Party member = 1; other = 0	0.017	0.130
	Mother's work status (a90d)	None = 0; agricultural = 1; non-agricultural = 2	0.886	0.381
	*Lifestyle*			
	Leisure reading (a3005)	Never = 0; several times a month = 1; several times a day and a week = 2	0.141	0.443
	Social intercourse (a3007)	Never = 0; several times a month = 1; several times a day and a week = 2	0.488	0.705
	Cultural activity (a3012)	Never = 0; several times a month = 1; several times a day and a week = 2	0.679	0.923
	Fitness exercise (a3009)	Never = 0; several times a month = 1; several times a day and a week = 2	0.603	0.851
	*Spouse status*			
	Spouse's work unit type (a87)	Social organizations or no work = 1; other = 2	1.593	0.491
	Spouse's education level (a72)	None = 0; primary school = 1; junior high school = 2; senior high school = 3; undergraduate = 4; postgraduate = 5	1.966	1.160
	Spouse's political status (a73)	Party member = 1; other = 0	0.126	0.332
Control variables	Female age	Actual age at the time of the investigation = 2015-a301	50.018	16.702
	Female political status (a10)	Party member = 1; other = 0	0.045	0.208
	Female marital status (a69)	Unmarried = 0; other = 1	0.928	0.258
	Female education level (a7a)	None = 0; primary school = 1; junior high school = 2; senior high school = 3; undergraduate = 4; postgraduate = 5	1.702	1.305
	Female work status (a58)	None = 0; agricultural = 1; non-agricultural = 2	0.806	0.861
	Female household registration type (a18)	Agricultural = 1; non-agricultural = 2	1.312	0.464
	Female sense of social equity (a35)	Completely unfair = 1; completely fair = 5 (increase in order)	3.194	0.995
	Housing property rights (a121)	Owned = 1; other = 0	0.358	0.479
	Female family economic class (a64)	Well below average = 1; well above average = 5 (increase in order)	2.622	0.702
	Female family class at 14 years of age (a434)	Lower class = 1; middle class = 2;upper class = 3	1.381	0.527

*In this table, the code in brackets below each index represents its corresponding question number in CGSS2015*.

#### Explained Variables

The explained variables mainly reflect the female's class identification, that is, the response to the following question in the CGSS2015 questionnaire: “What level do you think you are currently at?” To respond to this question, in the questionnaire, the respondents are presented a total of 10 options: a response with a value of 1 indicates the bottom level, and a value of 10 indicates the top level; the numbers in the middle increase as the level increases. The higher the number for the response is, the higher the class the women think they belong to is, and a higher number also indicates that they attach more importance to and recognize their social status. To refine and stratify the explained variables, the responses in which participants selected options 1–3 are grouped into the lower class and are assigned a value of 1; responses of 4–7 are grouped into the middle class and assigned a value of 2; responses of 8–10 are grouped into the upper class and assigned a value of 3. Through statistical analysis, the average value of female class identification is found to be 1.749, and the standard deviation is 0.485, which indicates that most women's class identification is lower than the middle class.

#### Explanatory Variables

The explanatory variables are divided into four aspects: gender consciousness, the mother's intergenerational influence, lifestyle, and spouse status influence.

**(1) Gender consciousness**. In the CGSS2015 questionnaire, there are five items about gender consciousness. The conceptual focus of each of the five items and the corresponding item text are as follows: gender division of labor consciousness, represented by the statement “Do you agree that men focus on career, women focus on family?” Gender ability consciousness, represented by the statement “Do you agree that male ability is naturally stronger than female ability?” Marriage consciousness, represented by the statement “Do you agree that having a good marriage is more important than having a good job?” Gender discrimination in employment, represented by the statement “Do you agree that female employees should be fired first in an economic downturn?” And housework distribution consciousness, represented by the statement “Do you agree that husband and wife should share the housework equally?” All these items reflect women's views on gender equality. For example, if women agree with the idea that “men's natural abilities are stronger than women,” they may feel inferior and dare not fight for their due rights, and even be less able to correctly recognize their social class (Tian et al., 2007). The participants are asked to respond to the above questionnaire items by answering as follows: if the respondents totally disagree, relatively disagree, are indifferent to agreeing, relatively agree, or fully agree with the item statement, they respond by assigning a value of 1, 2, 3, 4, or 5, respectively. The average values for the gender division of labor consciousness, marriage consciousness, and housework distribution consciousness were between 3 and 4, indicating that these three concepts were relatively neutral among female investigators. However, the average value of gender ability consciousness was 2.934, and the average value of gender discrimination in employment was 1.818, which indicates that most women do not agree with the view that “men are born with strong ability” and “women are discriminated against in employment.”

**(2) Mother's intergenerational influence**. If the mother has always been in a low position in the family or the mother's self-experience has intentionally or unintentionally set up a low female image for the children (especially the daughter), it will affect the class identification of the next generation. Class identification is closely related to education, political status, and work unit. Therefore, in the CGSS2015 questionnaire, three items focusing on the mother's intergenerational influence are included, namely, “What is the highest education level of the mother?” “What is the political status of the mother?” and “What is the employment status of the mother at the age of 14?” These three items represent the main background of the previous mother's generation. Significantly the mother's employment status may affect the family's income and even the status in children's hearts, and indirectly affect the shaping of children's female class identity values. For the mother's highest education level, the response values are assigned as follows: no education is assigned a value of 0; private school and primary school education are assigned a value of 1; junior high school education is assigned a value of 2; vocational high school, senior high school, secondary school, and technical school education are assigned a value of 3; undergraduate and specialty college education are assigned a value of 4; and postgraduate education and above are assigned a value of 5. According to the data statistics, the average of the highest education level of the mothers is 0.595, which indicates that the mother's education level is mainly primary school or no education. According to the calculation of the time, the period in which the mothers received education was mainly from 1950 to 1980, which was a period in which education was not popularized. The survey results confirm this fact regarding the unpopularity of education during that period as being basically true. For the political status of mothers, the “Party member” response is set to 1, and the others are set to 0: the average response value is 0.017, indicating that the majority of mothers are not party members. Through statistical data, it is found that the number of female respondents whose mothers are not party members is 4096. The employment status of mothers at the age of 14 will also affect the employment identification of the next generation. The value of no job is assigned as 0, that of agricultural work is assigned as 1 and that of non-agricultural work is assigned as 2. The average value of this index is 0.886, which indicates that most of the mothers of the women surveyed are engaged in agricultural work.

**(3) Lifestyle**. In the questionnaire of CGSS2015, four items are selected to represent the four aspects of lifestyle: the frequency of leisure reading, social intercourse, cultural activities, and fitness exercises, because now people pay more attention to the quality of life than before. If they carry out fitness exercises, social intercourse, and other activities, it also proves that their quality of life has increased, and the objective social class has improved (Chen and Lin, 2016). The subjective social class identity will be affected by the objective social class, so the lifestyle affects the objective social class, affecting the subjective social class identity. The answers to the questions for these items are as follows: every day, several times a week, several times a month, several times a year or less, and never, which are assigned a value of 1, 2, 3, 4, and 5, respectively. For the convenience of statistics, since “never” and “several times a year or less” mean that they have little impact on life, these responses are assigned a value of 0; “several times a month” is assigned a value of 1; and “several times a week” is assigned a value of 2. The leisure reading factor was represented by “participating in cultural activities, such as listening to concerts, watching performances, and exhibitions;” the respondents answers had an average value of 0.141, indicating that most women hardly have time to engage in leisure reading. The social factor was represented by “meeting with friends,” which had an average response value of 0.488. The factor of cultural activities was represented by “surfing the Internet,” and the average response value was 0.677. The factor of fitness exercise was represented by “taking part in physical exercise,” and the average response value was 0.602. The average value of the latter three factors fluctuated up and down 0.5, indicating that the frequency of the latter three factors was less than several times a month, but a response indicating that the respondent had never participated in these activities was never given.

**(4) Spouse status**. For most Chinese women, marriage has a great impact on their outlook on life and values. To study how much influence a spouse has on female class identification, it is necessary to screen the women's marital status. One of the questions about marital status in the CGSS2015 questionnaire is “What is your current marital status?” Women who choose “unmarried” are asked questions related to their parents, and the sample data of women with spouses are automatically screened out through stata15.0 software. The literature review reveals that the main factors related to female class identification are the spouse's education level, political status, and work unit type. Similar to the research on the mother's intergenerational influence, these three factors play an essential role in the social status of female spouses. In the traditional Chinese concept, the social status of female spouses will also affect women's judgment on their social class. The evaluation of females' spouse education level is the same as that of mother's intergenerational influence research, with an average of 1.966, indicating that most women's spouses have basically a primary or junior high school education. The political status of the females' spouses is the same as before, with an average value of 0.126, indicating that most spouses are not party members. In the questionnaire, for responses to questions regarding the work unit type of the females' spouses, the responses indicating the spouse worked in the party and government, enterprises, institutions and the army were assigned a value of 2; responses indicating social organizations and no work were assigned a value of 1. The average response value was 1.593, indicating that the working units of female spouses were mainly concentrated in the party and government, enterprises, institutions, and the army.

#### Control Variables

The control variables are mainly the individual factors influencing female class identification: the female's age, political status, marital status, education level, work status, the household registration type, social equity, housing property rights, family economic class, and family class at 14 years old. Among them, age is a continuous variable. Taking the survey year 2015 as the time point, the age of the respondents in 2015 was calculated. After a series of variables such as political status and marital status are selected, the values are assigned according to Table 1. In particular, this paper selects the property rights index of the house as the control variable, because the Chinese people think whether the house property certificate contains the owner's name is an important feature of family status. The age of 14 is an important period of human brain development (Hicks et al., 2015), as the family's class at the women's age of 14 will deeply affect the surveyed women's view of hierarchy. Through the data statistics, we found that the average age of the investigated women was 50.018, and the standard deviation reached 16.702, indicating that the age difference of the investigated women was very large. The average value of the women's political status was 0.045, indicating that women who were not party members accounted for a large proportion of the respondents. The average marital status of women was 0.928, indicating that unmarried women occupied a small proportion of the respondents. The average education level of women was 1.792, which indicates that most of the women surveyed had a primary or secondary school education. The average value of women's work status was 0.806, which indicates that most women's jobs are non-agricultural, a finding that is more in line with the current main working conditions. The majority of the workers are employed in non-agricultural industry, as the employment in the agricultural industry is gradually decreasing. The average value of the female household registration type was 1.312, which indicates that female respondents have a disposition to agricultural household registration. The average value of the women's sense of social justice was 3.194, which is relatively balanced. The average value of the women's housing property rights was 0.358, indicating that most of the surveyed women did not own their own housing property rights. The average value of the female's family economic status was 2.662, i.e., close to 3, which indicates that most women think their family economic status belongs to the middle or lower class. The average family class of women at the age of 14 was 1.381, indicating that most of the surveyed women considered their family class to be middle or lower class at the age of 14.

## Model Construction and Result Analysis

### Model Construction

Since quantifying the female stratum requires an ordered variable, we chose the ordered logit model for the analysis. After the data were processed, the multicollinearity test of the data was first carried out (Yu et al., 2015; Cavalletti and Corsi, 2018). According to the variance inflating factor (VIF), the larger the value is, the more serious the multicollinearity between explanatory variables is. Generally, when the value of VIF≥10, it means that there is serious multicollinearity between explanatory variables and other explanatory variables. When the value of VIF is closer to 1, it means that the multicollinearity is weaker. In this paper, the mean value of multicollinearity is 1.39, and the multicollinearity among the variables is <3, thus, there is no multicollinearity in each index. Therefore, we can analyze the influence of each index variable on female class identity. The formula is as follows:

(1) $$\begin{array}{r} Y_{i}^{*}=\beta X_{i}+\varepsilon_{i} \end{array}$$

Among the variables, $Y_{i}^{*}$ is the potential female class identity, *X*_*i*_(*i* = 1, 2, …, n) denotes the variables affecting the female class identity, β is the parameter to be estimated, and ε_*i*_ is the random disturbance term. The corresponding relationship between the unobservable potential variable $Y_{i}^{*}$ and the observable variable *Y* is as follows:

(2) $$\begin{array}{r} Y=\{\begin{array}{c} 1,\\ 2,\\ 3, \end{array}\begin{array}{c} \;Y_{i}^{*}\leq \mu_{1}\\ \mu_{1}<\;Y_{i}^{*}\leq \mu_{2}\\ \;Y_{i}^{*}>\mu_{3} \end{array} \end{array}$$

Among them, the actual observed female class identity is *Y*, with values of 1, 2, and 3 indicating the lower, middle and upper levels, respectively; μ_1_ and μ_2_ are the cutting points, both of which are parameters to be estimated, and μ_1_ < μ_2_.

### Explanatory Variables and Female Class Identification

To study the influencing factors of female class identification from different perspectives, this paper establishes five different models: model 1 is only the control variable; model 2 adds the “gender consciousness” factor to model 1, model 3 adds the “mother's intergenerational influence” factor to model 2; model 4 adds the “lifestyle” factor to model 3; and model 5 adds the “spouse status” factor to model 4. Through the results of each model, we can see the significant characteristics of each index variable.

### Results Analysis

Through the use of different research angles to study the influencing factors of female class identification, the results in Table 2 are obtained. Therefore, the results of each model can be analyzed separately.

**Table 2:** Analysis of factors affecting female class identification. *Source*: Author's own elaboration from the models' results.

**Independent Variables**	**Model 1**	**Model 2**	**Model 3**	**Model 4**	**Model 5**
**Gender consciousness**					
Gender division of labor consciousness		0.111^***^ (0.039)	0.120^***^ (0.041)	0.115^***^ (0.042)	0.041 (0.068)
Gender ability consciousness		0.028 (0.038)	0.027 (0.041)	0.028 (0.041)	0.059 (0.067)
Marriage consciousness		−0.098^***^ (0.036)	−0.099^***^ (0.038)	−0.102^***^ (0.038)	−0.053 (0.064)
Gender discrimination in employment		0.093^**^ (0.040)	0.107^**^ (0.043)	0.110^**^ (0.043)	0.132^*^ (0.079)
Housework distribution consciousness		0.027 (0.038)	0.016 (0.041)	0.007 (0.042)	0.014 (0.071)
**Mother's intergenerational influence**					
Mother's education level			−0.072 (0.059)	−0.072 (0.059)	−0.900 (0.092)
Mother's political status			0.173 (0.342)	0.175 (0.343)	0.094 (0.495)
Mother's work status			0.131 (0.106)	0.133 (0.107)	−0.019 (0.178)
**Lifestyle**					
Leisure reading				−0.140 (0.091)	−0.207 (0.148)
Social intercourse				0.030 (0.059)	−0.191^*^ (0.105)
Cultural activity				0.016 (0.062)	−0.126 (0.096)
Fitness exercise				0.076 (0.051)	0.182^*^ (0.089)
**Spouse status**					
Spouse's work unit type					0.142 (0.095)
Spouse's education level					0.267^*^ (0.144)
Spouse's political status					−0.077 (0.237)
**Control variables**					
Female age	0.005 (0.003)	0.004 (0.003)	0.001 (0.003)	0.001 (0.004)	−0.002 (0.008)
Female political status	0.313 (1.953)	0.296 (0.197)	0.320 (0.213)	0.308 (0.212)	0.419 (0.375)
Female marital status	0.387^**^ (0.160)	0.361^**^ (0.164)	0.356^**^ (0.172)	0.344^**^ (0.174)	
Female education level	0.940^**^ (0.042)	0.121^***^ (0.044)	0.127^***^ (0.048)	0.111^**^ (0.050)	0.042 (0.097)
Female work status	0.021 (0.045)	0.033 (0.046)	0.034 (0.049)	0.030 (0.050)	0.132^*^ (0.778)
Female household registration type	0.079 (0.092)	0.110 (0.095)	0.176^*^ (0.104)	0.165 (0.106)	−0.068 (0.180)
Female sense of social equity	0.233^***^ (0.036)	0.212^***^ (0.037)	0.233^***^ (0.040)	0.230^***^ (0.040)	0.221^***^ (0.069)
Housing property rights	0.095 (0.075)	0.101 (0.078)	0.099 (0.083)	0.115 (0.084)	−0.007 (0.150)
Female family economic class	1.112^***^ (0.055)	1.097^***^ (0.057)	1.136^***^ (0.062)	1.122^***^ (0.062)	1.209^***^ (0.118)
Female family class at 14 years old	1.552^***^ (0.086)	1.590^***^ (0.089)	1.569^***^ (0.095)	1.562^***^ (0.096)	1.696^***^ (0.165)
*R*^2^	0.184	0.186	0.191	0.190	0.203

*In this table, the numbers in brackets indicate the standard deviation; the numbers outside the brackets represent the explanatory variables, and the significance meaning is attached after them*.

*, **, and

*** *denote significance at the 10, 5, and 1% levels, respectively. The blank in the table indicates that the indicator variable is not used in the corresponding model*.

#### Gender Consciousness and Female Class Identification

Model 2 is based on the control variables of model 1, to which various factors of gender consciousness are added in order to carry out the research. The gender division of labor consciousness is found to have a significant positive effect at the level of 1%, which indicates that the more women agree with the view that “men focus on career, women focus on family,” the higher the women's class consciousness. The main reason for this is that most Chinese women are more traditional. When their husbands are successful in their careers, women are willing to be their supporters. The more successful their husbands are, the greater they feel that their own class will be promoted. At the level of 1%, marriage consciousness has a significant negative impact on class consciousness, which indicates that the more women accept the view that “having a good marriage is more important than having a good job,” the lower their own class consciousness. Especially in the marriage market, many women hope to change their own class through marriage, and such women generally have low self-recognition. Gender discrimination in employment is significant at the level of 5%; that is, the more women agree that “female employees should be fired first when the economy is depressed,” the higher the women's class consciousness, which is inconsistent with the original hypothesis. In conclusion, hypothesis H1 of gender consciousness has been partially verified.

#### Mother's Intergenerational Influence and Female Class Identification

Model 3 is based on the addition to model 2 of various factors of the mother's intergenerational influence. Based on a literature review and the psychological definition of a family relationship, the main factors selected in this paper are as follows: the mother's highest education level, political status, and work status. However, these three indicators are not significant. Therefore, in this study, we refuse to assume H2 and find that mothers have no influence on the class identification of the next generations of women in the family. The reason may be that with the gradual popularization of modern social views and the increasing number of female workers, the acceptance of the former view that “men are in charge of the outside world, and women are in charge of the interior” or “housewives” has now been reduced. Therefore, the working status of mothers has little impact on the female class identification of the next generation.

#### Lifestyle and Female Class Identification

Model 4 is based on the addition of various factors of lifestyle to model 3. In model 4, the factors of lifestyle are not significant, which shows that the improvement of lifestyle has no influence on female class identification. However, note that in model 5, after considering the influence of the female's spouse, the social activities show a significant negative influence at the 10% level, while fitness exercise shows a significant positive effect at the 10% level. This may be because women will put more energy toward family after marriage. If women's social activities are too frequent, it is not conducive to family harmony and can thus affect women's class identification. The positive effect of fitness exercise may be because the couple's fitness exercise can promote the relationship between husband and wife but can also increase the happiness and sense of gain in women's daily life such that it can enhance women's class identification. Therefore, hypothesis H3 is partially proved, indicating that lifestyle has some influence on female class identification.

#### Spouse Status and Female Class Identification

Model 5 establishes a model of a married female's class identification. From the model, it can be found that the education level of a spouse has a significant effect on female class identity at the level of 10%. In reality, when women are looking for marriage partners, the partners' education level is a very important choice factor; this view can be proved by the finding that the spouse's education level has an important impact on the married women's class identification. However, there was no significant effect of the spouse's work unit type and political status on the women's class identification. Therefore, hypothesis H4 has been partially proved.

#### Female Personal Characteristics and Female Class Identification

In model 5, we can clearly find the significance of each control variable index. The working status of women is positively significant at the level of 10%, which indicates that the social stratum identity of women in non-agricultural work is higher than that of women in agricultural work or unemployment. The women's sense of social justice is significant at the level of 1%, which indicates that the women's feeling regarding the fairness of social justice is very important for female class identification. The economic status of the women's family is positively significant at the level of 1%, which proves the importance of family economic status to women. The better the family economic status is, the higher the class identification of women. The family class of women at the age of 14 is significant at the level of 1%, as the age of 14 is a critical period for the formation of one's values. Therefore, the family class at the age of 14 will affect the women's future class identification, which is a finding consistent with the statistical results.

## Conclusion And Discussion

### Conclusion

With the continuous progress of social civilization, gender equality has always been the goal advocated and pursued by modern society (Zajacova, 2006; Nyman et al., 2018). The countries in the world have made great progress in improving laws and regulations and protecting the women's status (Besamusca et al., 2015; Koburtay et al., 2020). However, have the women's own values truly progressed with the development of society? Can a woman recognize herself based on the feelings from the bottom of her heart, not from the external evaluation or her husband? When the female individual subjective social class identity is higher, it shows that they are confident about their situation, considering a higher social class and a higher degree of identity and dependence on society. The more critical practical significance is that the level of women's social subjective cognition is directly related to the effective solution of many social problems, the likelihood of influence on the relationship with other social members, and the resolution of social contradictions (Hudson, 2015; Cole, 2020).

Based on the CGSS2015 data, in this paper, a female class identity framework is constructed and the ordered logit model is used to analyze the influence of gender consciousness, mother intergenerational influence, lifestyle, and spouse status on the women's class identification. The conclusion is as follows: (1) the factors of gender consciousness have a significant impact on female class identification; (2) the factors of the mother's generation have no influence on female class identification; (3) on the premise that women have spouses, the factors of lifestyle have some significant effects on female class identification; (4) the status of the spouse, especially the education level of the spouse, has a significant positive impact on female class identification.

### Discussion

The main contribution of the study is that, compared with previous studies (Zipp and Plutzer, 1996; Hudson, 2015; Leicht et al., 2017; Zang, 2020), this paper does not only consider the influence of a single factor, but also comprehensively considers several essential factors that may affect the formation of class concept in women's personal growth experience to restore the process of the construction of women's class concept. The results of the model further not only prove that gender consciousness and spouse status have the significant effects on women's class identity, but also indicate that mother intergenerational influence has little impact on women's class identity. This is also a vital breakthrough compared with previous studies. The influence of mother on daughter may not be as significant as people think. The shaping of postnatal concept is more important, highlighting the importance of social guidance and the formation of ethos.

However, there are some limitations in this study. This paper only considers the whole class concept of women. There are significant differences in the growth environment and education situation between urban and rural women in China, which is also the direction of our future research. Besides, there are still insufficient indicators in this paper. For example, there are only three indicators related to the mother's intergenerational influence and spouse status, which may not fully explain. And our research takes women's marriage as the end of the research period, but women may also experience some changes in their middle and old age. If we can consider a more extended period, that is, to study all the influencing factors of women's whole life class identity, the interpretation of women's class research will be more accurate. The last limitation is that we only used CGSS data, and the regional differences factors are not taken into account. In the future, we can use the relevant international public data to conduct more comprehensive research, especially compare the differences in factors influencing female class identification between different countries and regions.

From the conclusion of this study, there are many factors that affect the women's class identification. Therefore, it is necessary to improve the women's ability and strengthen the women's consciousness of power. It is suggested that women's class identification should be promoted from the following aspects.

**(1) Protecting women's basic rights and interests**

Much discrimination toward women still exists in society; one obvious example is the gender discrimination in the process of job hunting (Uunk, 2015; Andersson and Harnois, 2020; Kuhn et al., 2020; Takahashi et al., 2020). Some companies do not recruit female job seekers even though there are no explicit regulations for recruitment. For women who have given birth or are about to give birth, the company will give the opportunities that originally belonged to them to male employees or new employees, which not only increases the reproductive pressure of women but also increases the work pressure of women. Therefore, women should be given tolerance and equal treatment in vulnerable periods, such as illness, childbirth or family changes. Therefore, we should also improve the relevant laws and policies to protect the rights and interests of women in employment and entrepreneurship. From the perspective of the overall interests of the society, if, as mothers, women have independent work, they can not only gradually get rid of their dependence on their spouses but also strengthen their own class identification. Especially for the next generation, confirming the so-called adage “teaching by example is better than words,” the example these mothers will create better. In 2020, China's newly revised “Civil Code” has strengthened the superior protection of women's rights and interests, provided more trustworthy and reliable legal protection for women, and rectified all kinds of adverse social phenomena affecting women. In particular, the law includes such contents as rural women's right to land (Han et al., 2019; Doss and Meinzen-Dick, 2020), opposition to sexual harassment and privacy protection, and recognition of women's family contribution. These newly drafted legal provisions have built a solid legal barrier for promoting gender equality and women's all-around development. While emphasizing the equal civil legal status of men and women, the superior protection of women's rights and interests has been strengthened. For the protection of women's class, the law has made some progress, but it still needs women's awareness and fight for their rights and interests.

**(2) Promoting women's independent education**

Real equality is the equality recognized by one's own heart. Some women firmly believe in gender inequality and therefore enjoy the goods or the treatment that they receive from their weak position as women. However, in fact, real equality comes from women's deep recognition of themselves. When they have the confidence, they can fight for the rights they deserve and will not let others take advantage of women's status. Therefore, in the new era, we should implement the concept of women's self-reliance, and women should pursue a completely independent life. In view of this, the state can also carry out various training lectures in order to strengthen women's skills and can conduct public welfare activities in order to improve women's cultural literacy so that women can have a greater sense of participation and happiness in society.

**(3) Enrich national entertainment activities**

The grass-roots government can better promote the participation of all people in entertainment, especially the collective entertainment activities involving the family as a unit. These activities play a very important role in promoting family harmony and women's class identification. In addition, these types of activities also effectively enable women to avoid the role of only being a housewife and increase their sense of social participation, the lack of which can lead to their disconnection from society and then the reduction of their social identity.

**(4) Improving women's education**

With social progress, the level of development of rural areas will gradually catch up with that of the cities (Zajacova, 2006; Fagertun, 2017; Dong et al., 2019; Baylina and Rodó-Zárate, 2020). However, in some surveys, we can still find the differences in women's status between urban and rural areas (Pedersen, 2016; Andersson and Harnois, 2020). At present, there are still many girls who do not have the opportunity to study. Therefore, in the process of promoting women's status, rural areas are still the most important and the most difficult bottleneck to break through. In rural areas, we should not only strengthen the legal protection of women and change rural women's views on gender equality but also protect the right of rural girls to receive education and promote rural women's employment opportunities (Wang et al., 2013).

Gender equality is advocated by many countries in the world (Nyman et al., 2018; Gupta et al., 2019; Łapniewska, 2019; Koburtay et al., 2020). However, little attention has been paid to women's own class identification, especially the influence of women's personal growth experience on their own class identification. Moreover, the degree of this influence has not been fully studied. Based on women's personal growth experience from youth to middle age, to study the factors that dominate female class identification, this paper creatively constructs a female class identification framework from five aspects: the mother's intergenerational influence, female personal characteristics, lifestyle, gender consciousness, and spouse status. This study can provide a theoretical and practical reference for further promoting gender equality, women's own class identification and women's psychological health.

## Data Availability Statement

The raw data supporting the conclusions of this article will be made available by the authors, without undue reservation.

## Author Contributions

PC and JZ: conceptualization, methodology, formal analysis, writing–original draft, and writing–review and editing. PJ: conceptualization, formal analysis, writing–review and editing, funding acquisition, and project administration. ZZ: methodology and funding acquisition. All authors: contributed to the article and approved the submitted version.

## Conflict of Interest

The authors declare that the research was conducted in the absence of any commercial or financial relationships that could be construed as a potential conflict of interest.
